# Integration analysis of single-cell and spatial transcriptomics identifies prognostic genes associated with neddylation in colorectal cancer

**DOI:** 10.1007/s12672-025-04340-y

**Published:** 2025-12-24

**Authors:** Ziming Zhu, Xinyue Zhang, Song Wang, Yunsi Huang, Xuedong Han, Dongping Lai, Xin Yao, Weixuan Lan, Hui Nong, Wenbin Zeng, Yanhua Mo, Ri’an Xu, Tao Zhang

**Affiliations:** 1 Department of Gastroenterology, Ruikang Hospital Affiliated to Guangxi University of Chinese Medicine, Nanning, China; 2 Guangxi University of Chinese Medicine, Nanning, China; 3 Department of Gastroenterology, Guidong People’s Hospital of Guangxi zhuang Autonomous Region, Wuzhou, China

**Keywords:** Colorectal cancer, Neddylation, Single-cell analysis, Spatial transcriptome, Epithelial cells

## Abstract

**Background:**

Neddylation modifications in immune and tumor cells are linked to poor tumor prognosis. This study identifies prognostic genes associated with neddylation-related genes (NRGs) in colorectal cancer (CRC) using single-cell and spatial transcriptome (ST) sequencing, aiming to advance CRC treatment strategies.

**Methods:**

Datasets included TCGA-CRC (training/internal validation, 7:3 split), GSE28722 (external validation), GSE132257 (scRNA-seq), and GSE226997 (ST). Single-cell analysis annotated seven cell types, with epithelial cells identified as key. Differentially expressed genes (DEGs) from key cells [DEGs(sc)] and bulk analysis of TCGA-CRC [DEGs(bulk)] were intersected with 247 NRGs to yield candidate genes. Regression analyses screened prognostic genes for risk model construction, validated internally and externally. Pseudotime trajectory and ST mapping visualized gene expression, while molecular networks and drug predictions were generated.

**Results:**

In scRNA-seq dataset, seven cell types were annotated, and epithelial cells were the key cells. A sum of 32 candidate genes were obtained by intersecting 5,131 DEGs(sc)(key cells), 9,089 DEGs(bulk), and 247 NRGs to produce PSMD12, PSMB2, and FBXL5 as prognostic genes. Both prognostic risk model and nomogram model were predictive of CRC. At the ST samples, PSMD12 was expressed at a low level in all sections, whereas PSMB2 and FBXL5 were expressed at a slightly higher level in the sections. In addition, a lncRNA-miRNA-mRNA network and a drug-prognostic gene network were created, getting some potential drugs like bortezomib.

**Conclusion:**

A novel three-gene prognostic model for CRC was developed and validated, offering therapeutic insights through molecular networks and drug predictions.

**Supplementary Information:**

The online version contains supplementary material available at 10.1007/s12672-025-04340-y.

## Introduction

Colorectal cancer (CRC) is a widely recognized malignant neoplasm and is among the foremost contributors to cancer-related fatalities globally. According to the National Cancer Center of China, data from 2022 indicated that CRC was the fourth most prevalent cancer and the fifth leading cause of death among male patients, while it ranked third in incidence and fourth in mortality among female patient [1]. Although screening initiatives have demonstrated efficacy in decreasing both the incidence and mortality rates of CRC [2], it continues to be the second leading cause of cancer-related deaths and the third most frequently diagnosed cancer worldwide [3, 4], with a notable rise in early-onset CRC cases [5].Timely detection and intervention are crucial for enhancing survival rates and significantly lowering CRC incidence, as approximately 25% of patients are diagnosed at an advanced stage, and nearly 25%−50% of those with early-stage disease may progress to metastasis [6, 7]. Metastasis is the primary contributor to fatalities associated with cancer. Among the prevalent treatment modalities for this condition are chemotherapy and subsequent surgical intervention; however, these current therapies are unable to fully eliminate the disease. Patients diagnosed with oligometastatic disease following tumor resection and systemic therapy exhibit a 5-year survival rate of 40%, in contrast to those with metastatic colorectal cancer, who have a survival rate of approximately 20% [8]. Despite advancements in early detection, surgical interventions, chemotherapy, and targeted therapies, mortality rates continue to be elevated in certain countries due to the intricate nature of colorectal cancer (CRC) and the difficulties associated with managing metastatic CRC [9]. Furthermore, it is widely recognized that colorectal cancer (CRC) is a highly diverse malignant condition capable of metastasizing to various sites, including lymph nodes, liver, lungs, bones, and ovaries [10]. Nevertheless, the specific varieties of heterogeneous cancer cells found in primary colorectal cancer (CRC) and their mechanisms of metastasis to specific target organs are still not well understood [11]. The diversity of colorectal cancer (CRC) and the constraints of existing treatment approaches highlight the critical necessity for discovering new prognostic genes that could function as viable therapeutic targets.

Post-translational modification (PTM) denotes the biochemical processes that involve the addition or removal of chemical moieties to or from the amino acid residues of a protein subsequent to its synthesis, facilitated by enzyme-mediated reactions [12]. It can significantly alter the protein’s structure, stability, function, and localization [13], including phosphorylation, acetylation, methylation, ubiquitination, glycosylation, and so on [14, 15]. Neddylation is a distinct form of post-translational modification characterized by the covalent linkage of the ubiquitin-like protein Nedd8 (Neural precursor cell expressed, developmentally down-regulated 8) to the substrate protein [16]. It is integral to numerous biological mechanisms, including cell cycle regulation, DNA repair, programmed cell death, and signal transduction pathways [17, 18]. Recent investigations have demonstrated that numerous proteins within immune and neoplastic cells experience neddylation [19]. In the realm of tumor biology, neddylation proteins are likely implicated in the modulation of tumor cell survival, proliferation, invasion, and metastasis. Furthermore, elevated expression levels of numerous neddylation-associated proteins in tumors correlate with unfavorable patient outcomes, suggesting that neddylation may be a critical factor in tumor progression and metastatic dissemination [20]. In the tumor microenvironment, the presence and functionality of immune cells are modulated by a range of factors. Neddylation may impact the activity and longevity of immune cells, consequently affecting tumor immune evasion and the efficacy of immunotherapeutic strategies [21]. Crucially, in colorectal cancer (CRC), neddylation modifications may influence the quantity and functionality of immune cells within the tumor microenvironment, thereby significantly contributing to the pathogenesis and therapeutic responses associated with CRC [22]. Consequently, targeted therapeutic strategies against neddylation could emerge as novel treatment approaches for CRC. However, the mechanism of the involvement of neddylation in the pathological process of CRC remains to be further studied.

Single-cell RNA sequencing (scRNA-seq) is a technique utilized to quantify the expression levels of all genes within individual cells, thereby elucidating the heterogeneity of cancer cells in the context of the tumor microenvironment [23]. Nevertheless, the temporal and spatial resolution of the technology remains inadequate and is currently undergoing enhancements [24]. The emergence of spatial transcriptomics (ST) overcomes this limitation, as it has been employed to clarify the interactions between various cancer cell types and the tumor microenvironment [25, 26]. Thus, by combining single-cell sequencing’s gene expression profiling with spatial transcriptomics’ mapping of cellular interactions, we can unveil the complexity of biological systems.

This study combined scRNA-seq data, transcriptome data, ST data and neddylation relate genes, explores relevant key cells in different ecosystems, is designed to characterize cell types, cell-cell interactions, and key molecules, and screened prognostic genes related to neddylation modification in CRC. It analyzes the potential mechanisms of these prognostic genes in CRC, hoping to provide new insights for the diagnosis and treatment of CRC.

## Results

### There were seven cell types in scRNA-seq dataset

**Fig. 1:**
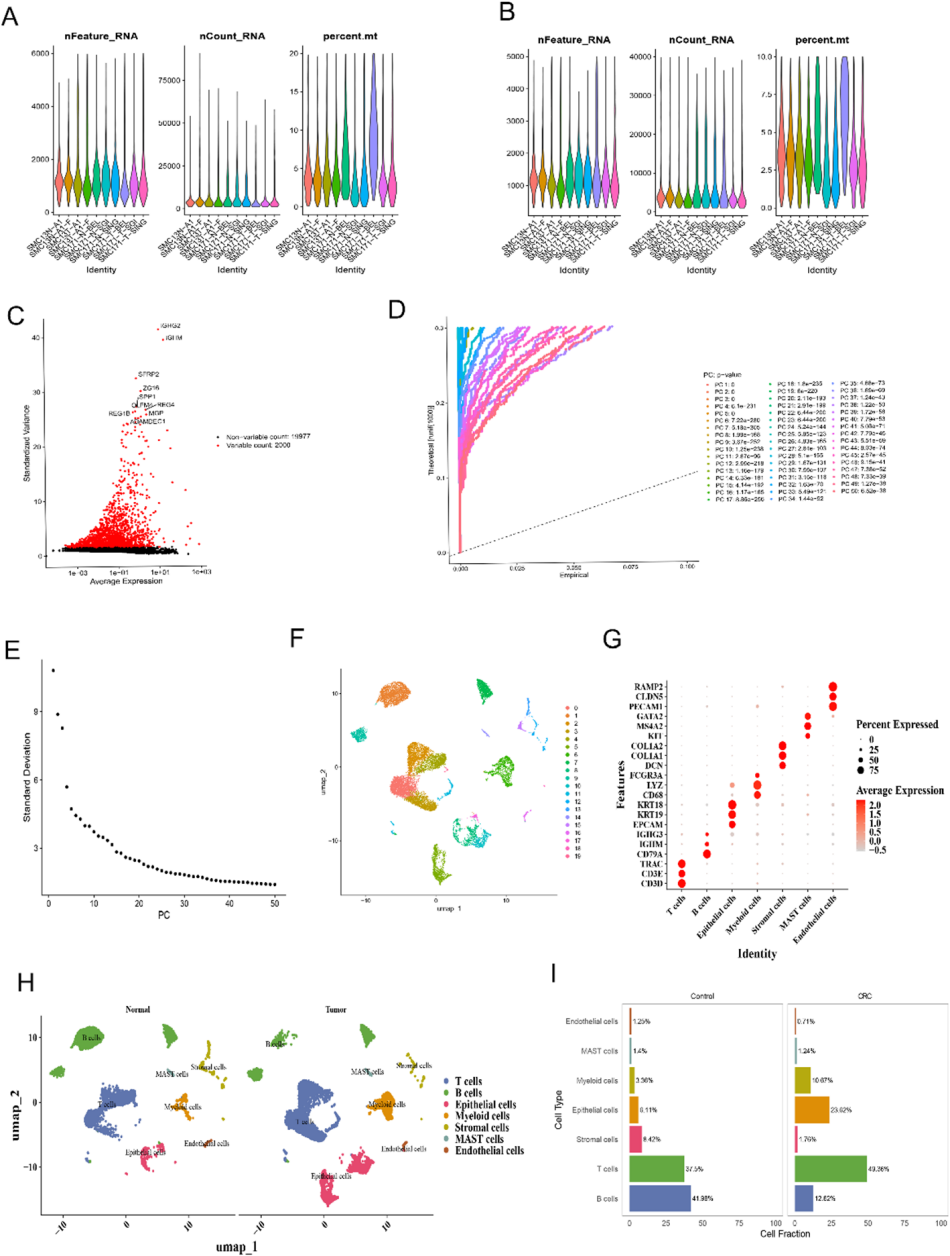
Single-cell RNA sequencing analysis comparing control and CRC samples.** A**,** B** Violin plots show nFeature_RNA, nCount_RNA, and percent.mt for different cell type samples before (**A**) and after (**B**) QC of single-cell data.** C** Highly variable genes in red, non-highly variable genes in black.** D**,** E** PCA elbow plots illustrating variance explained by principal components and the optimal number for analysis.** F** UMAP plot shows clusters of different cell types based on marker gene expression.** G** Dot plot of marker gene expression across cell types, with dot size shows percentage of cells and color show expression level.** H** UMAP plot comparing control and CRC samples, highlighting major cell types in both conditions.** I** Bar plot shows differences in cell type fractions between control and CRC samples

For scRNA-seq data, there were 18,409 cells before filtration, with 15,145 cells and 21,977 genes remaining after filtration (Fig. 1A, B). Then 2000 highly variable genes were chosen with labeling top 10, which were IGHG2, IGHM, SFRP2, ZG16, SPP1, REG4, OLFM4, REG1B, MGP, and ADAMDEC1 (Fig. 1C). The top 30 PCs were picked for subsequent analyses based on PCA inflexion and fragmentation plots (Fig. 1D, E). And 20 cell clusters were eventually generated and annotated into seven cell types, including T cells, B cells, epithelial cells, myeloid cells, stromal cells, mast cells, and endothelial cells, according to marker genes (Fig. 1F-H). The proportions of various cell types showed that T cells and B cells were more evenly distributed in control group, while T cells had the highest proportion in the CRC group (Fig. 1I).

**Fig. 2:**
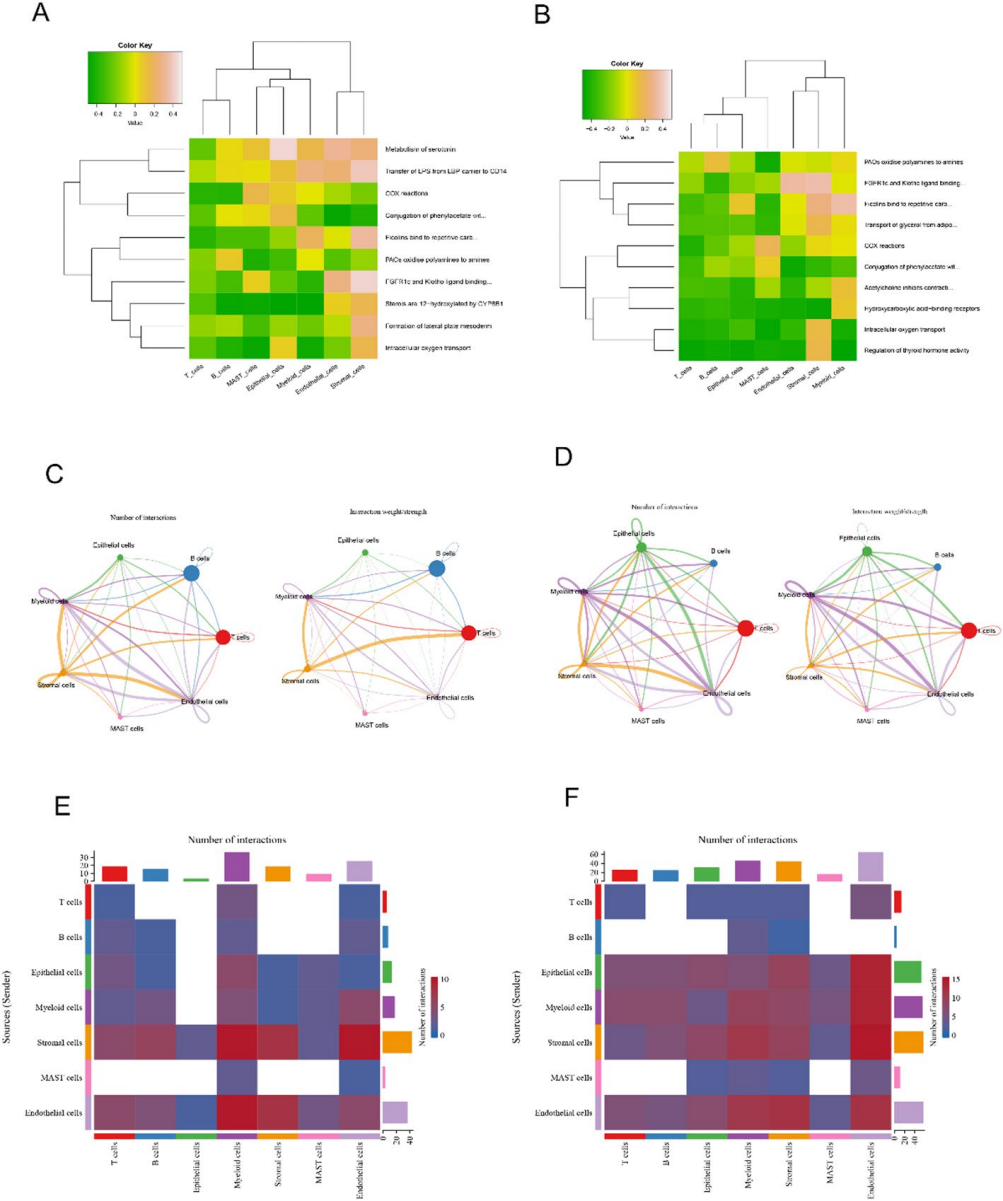
Comparison of GSVA enrichment and cellular interactions between normal and CRC samples. **A**,** B** GSVA heatmaps show scores in different cell types for the normal (**A**) and CRC (**B**) groups. The color intensity reflects the enrichment levels of key biological processes across cell types, with hierarchical clustering revealing relationships between pathways.** C**, **D** Network plots displaying predicted cell-cell communication interactions in normal (**C**) and CRC (**D**) groups. Each node represents a cell type, and the thickness of the edges indicates the strength of interactions. Larger nodes correspond to cell types with more interactions.** E**,** F** Heatmaps of the number of interactions between different cell types in the normal (**E**) and CRC (**F**) groups. Colors represent the interaction frequencies, with both sender (rows) and receiver (columns) roles highlighted for each cell type

GSVA results revealed that seven cell types in control group were mainly enriched into metabolism of serotonin, transfer of LPS from LBP carrier to CD14 and so on, while cell types in CRC group were mainly enriched for pathways such as PAOs oxidize polyamines to amines, FGFR1c and Klotho ligand binding and activation, and COX reactions (Fig. 2A, B). Communication analysis was also performed for seven cell types in both CRC and control groups. Stromal cells and T cells had higher communication in both number and strength in control group (Fig. 2C). In CRC group, myeloid cells and endothelial cells had the highest number and strength of communications between them (Fig. 2D). The heatmap even more clearly showed that in the control group stromal cells have strong communication with myeloid and endothelial cells, myeloid cells and stromal cells had strong communication strengths in CRC group(Fig. 2E, F).

### Altogether 32 candidate genes were connected to neddylation and CRC

**Fig. 3:**
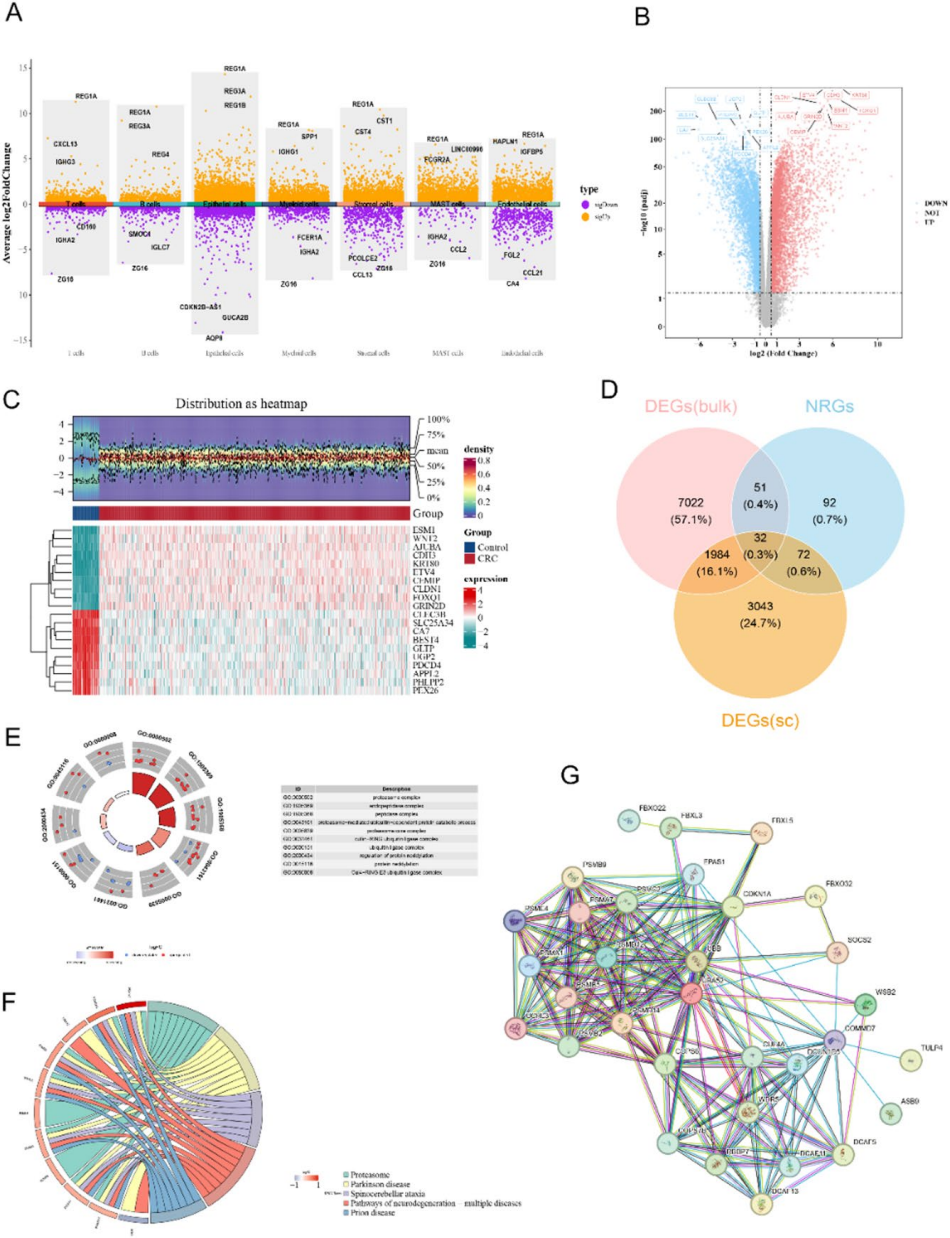
Candidate gene screened and enrichment analyse. **A** Differential gene expression across cells. Average log2 Fold Change (FC) on the y-axis, with yellow dots indicating upregulated genes and purple dots for downregulated genes. **B** Training set differential expressed gene analysis. Red denotes upregulated genes, and blue indicates downregulated genes. **C** Heatmap of the top 10 differentially expressed genes. **D** Venn diagram of 32 candidate genes intersected from Degs(sc)(key cells), Degs(bulk) and NRGs. **E** GO annotation of the 32 candidate genes. **F** KEGG pathway annotation of the 32 candidate genes. **G** PPI network of the 32 candidate genes

A total of 710 genes were over-expressed and 432 were under-expressed in B cells. In endothelial cells, 1288 genes were over-expressed and 487 were under-expressed. In epithelial cells, 3417 genes were over-expressed and 1714 were under-expressed. A sum of 1497 over-expressed genes and 77 under-expressed genes were produced in mast cells. In myeloid cells, 1539 genes were over-expressed and 443 were under-expressed. In stromal cells, 2824 genes were over-expressed and 560 were under-expressed. In T cells, 1000 genes were over-expressed and 336 were under-expressed (Fig. 3A). Of these, epithelial cells had the most DEGs and were therefore selected as key cells, containing 5131 DEGs(sc)(key cells). Meanwhile, totally 9089 DEGs(bulk) were detected in TCGA-CRC, of which 4,794 over-expressed and 4295 under-expressed in CRC group(Fig. 3B, C). A sum of 32 candidate genes connected to neddylation and CRC were obtained by intersecting 5131 DEGs(sc), 9,089 DEGs(bulk), and 247 NRGs (Fig. 3D). These candidate genes enriched in 167 GO terms, containing proteasome complex, endopeptidase complex, peptidase complex, etc. (Fig. 3E). They also enriched in 16 KEGG pathways like proteasome and spinocerebellar ataxia (Fig. 3F). In the end, the PPI network composed of candidate genes had a total of 178 relational interaction pairs (Fig. 3G).

### PSMD12, PSMB2, and FBXL5 were prognostic genes to develop risk model

**Fig. 4:**
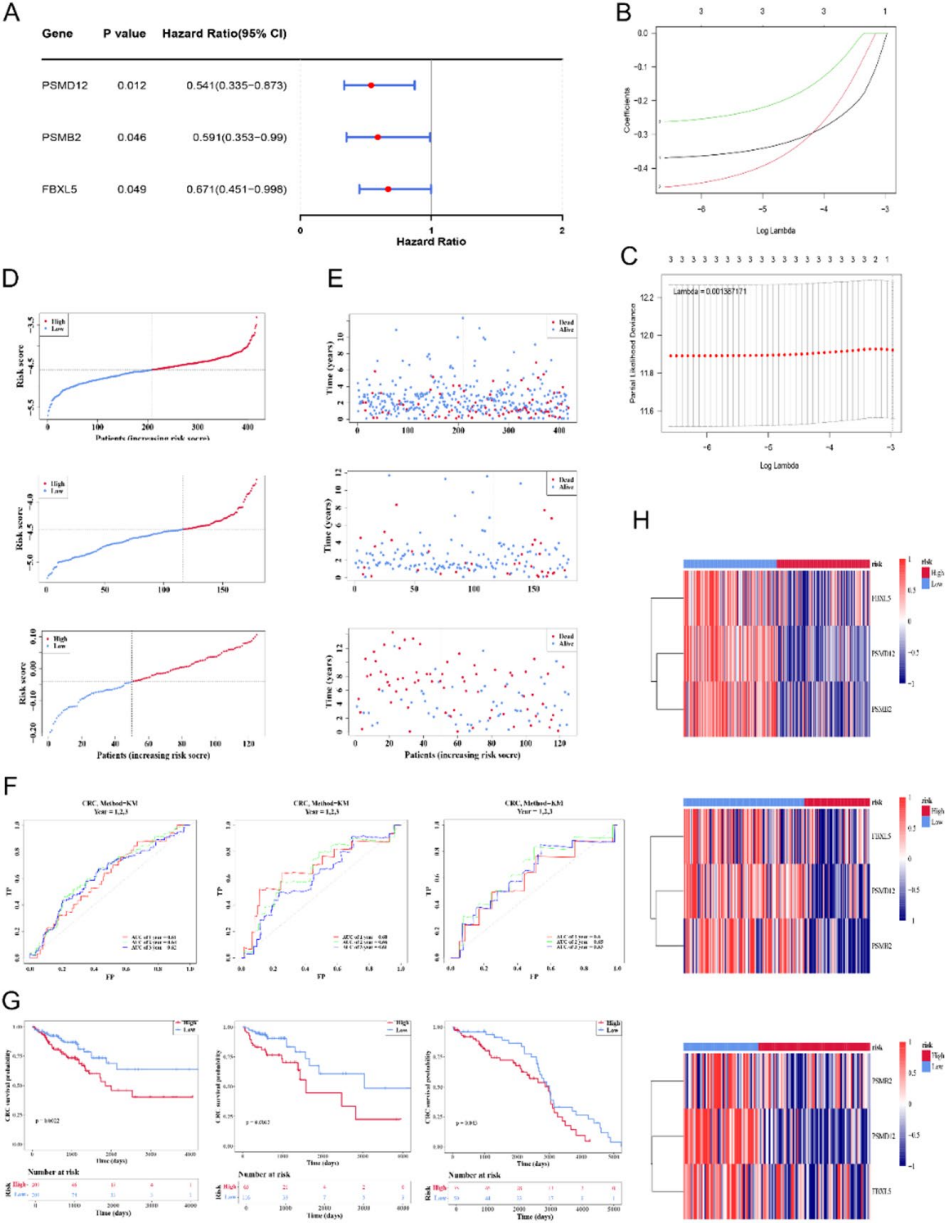
Evaluation and validation of the prognostic risk model in 3 datasets (training set, internal validation set, and external validation set). **A** Univariate Cox analysis of 32 candidate genes identified PSMD12, PSMB2, and FBXL5 as the prognostic genes. **B** LASSO coefficient profiles for different log(lambda) values, with the optimal lambda indicated. **C** Cross-validation for selecting the best λ value (0.001367171) in the LASSO model. **D** Risk score distribution and survival status for patients, showing higher mortality in the high-risk group. **E** Dot plots of patient survival times, comparing alive (blue) and dead (red) based on risk scores. **F** Time-dependent ROC curves evaluate the prognostic model’s accuracy at various time points. **G** Kaplan-Meier curves show survival differences between high- and low-risk groups. **H** Heatmaps of PSMD12, PSMB2, and FBXL5 expression in high- and low-risk groups

After univariate Cox analysis and PH test of 32 candidate genes, a pool of three genes was detected to be associated with prognosis of CRC (Fig. 4A; Table S2). The LASSO model fitted best when lambda was taken to 0.001367171, corresponding to a number of three prognostic genes (PSMD12, PSMB2, and FBXL5) (Fig. 4B, C). Risk score = − 0.3698842×PSMD12-0.4556811×PSMB2-0.2623736×FBXL5.

The prognostic risk model was evaluated and verified in three datasets. In accordance with the prognostic risk model, risk scores were calculated and all samples in each dataset were categorized into high-/low-risk cohorts based on their optimal thresholds. In the three datasets, there was a clear distinction between the risk scores of two risk cohorts (Fig. 4D). Survival of each sample was shown in each of the three datasets separately, which revealed that the risk scores were effective in demonstrating the prognosis of the patients with CRC (Fig. 4E). The risk model had a 1 year prognostic area under the ROC curve (AUC) of 0.61, a 2 year prognostic AUC of 0.64 and a 3 year prognostic AUC of 0.62 in training set; AUC values of 0.68, 0.66, and 0.61 for 1-, 2-, and 3 year in internal validation set; and accordingly, AUC of 0.60, 0.65, and 0.63 in external validation set (Fig. 4F). All AUC values were over 0.6, signifying that the risk model was more accurate in predicting CRC. Patients in low-risk cohort survived markedly better than those in high-risk cohort for all three datasets (*p* < 0.05) (Fig. 4G). Also, all three prognostic genes were lowly expressed in the high-risk cohort (Fig. 4H).

### Nomogrm model was valid and high-risk cohort was linked to tumor progression by GSEA

**Fig. 5:**
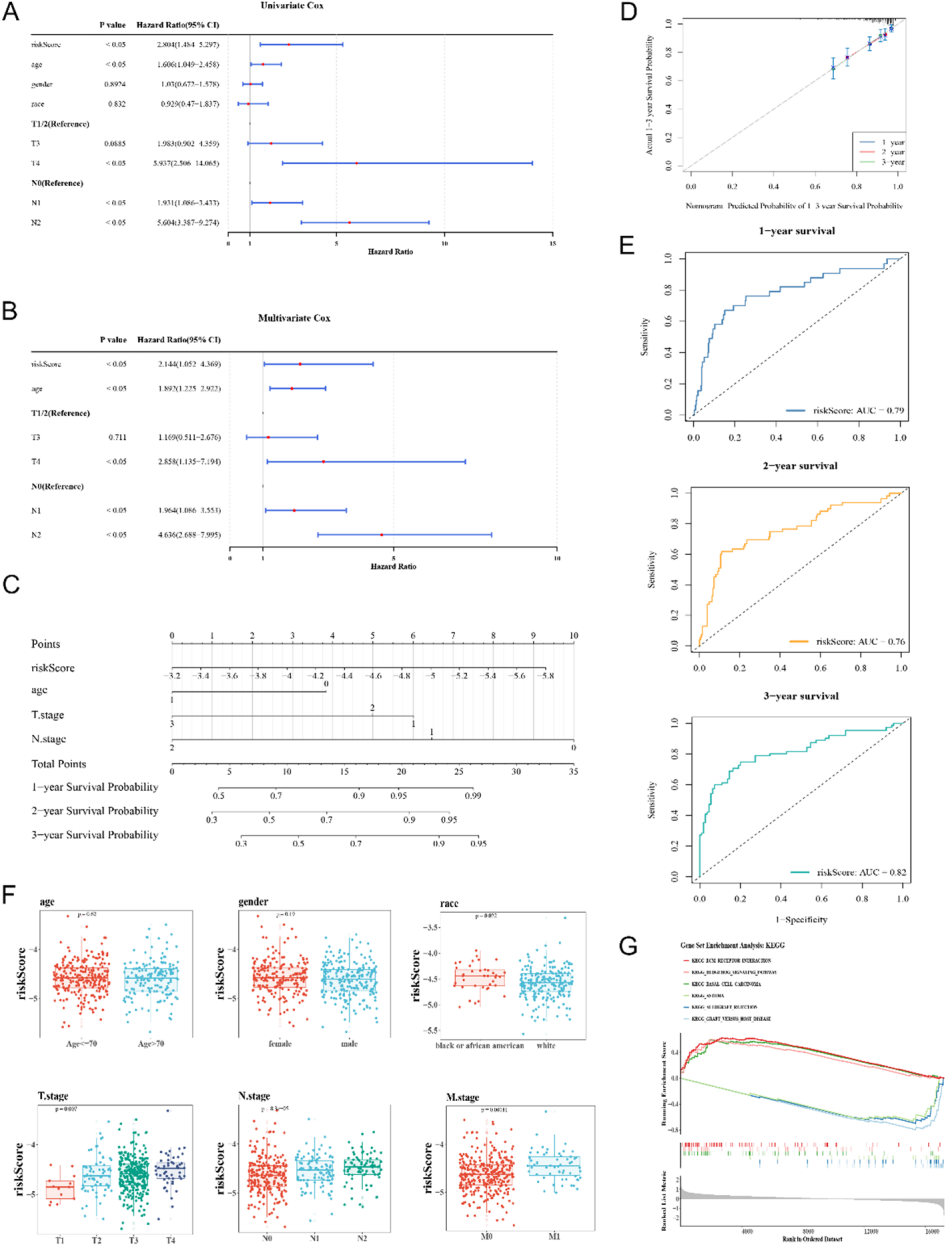
Prediction of prognostic nomogram and GSEA. **A** Univariate Cox regression analysis shows hazard ratios (HR) for risk score, age, gender, race, T stage, and N stage, with significant associations. **B** Multivariate Cox regression analysis adjusting for covariates, highlighting independent risk factors. **C** Nomogram predicting 1-, 2-, and 3 year survival probabilities based on risk score, age, and cancer stage. **D** Calibration plot comparing predicted vs. actual 1-, 2-, and 3 year survival probabilities. **E** ROC curves assessing the prognostic accuracy for 1-, 2-, and 3 year survival, with AUC values of 0.79, 0.76, and 0.82, respectively. **F** Box plots show the distribution of risk scores across different clinical and demographic factors. **G** GSEA plot shows enriched pathways based on risk score stratification

The final independent prognostic factors chosen through Cox analyses were risk score, age, T stage, and N stage (Fig. 5A, B; Table S3). A nomogram was constructed based on these factors, in which a higher total points summed by each factor represented a higher survival rate of CRC patients (Fig. 5C). Furthermore, calibration curve demonstrated that nomogram model predicted survival rates of CRC patients accurately, indicating that the model was valid (Fig. 5D). The AUC values of the nomogram exceeded 0.7 at 1-, 2- and 3-year, suggesting that the nomogram model presented excellent predictive power (Fig. 5E). Furthermore, the risk scores of CRC patients exhibited a statistically significant difference (*p* < 0.05) among race, T, M and N stage subgroups, respectively (Fig. 5F). Furthermore, the TCGA-CRC high-risk cohort was found to be significantly enriched in ECM-receptor interaction, hedgehog signaling pathway, basal cell carcinoma and so on, indicating a focus on tumor progression and cellular interactions, while the low-risk cohort demonstrated a significant enrichment in asthma, allograft rejection, graft-versus-host disease and so on (Fig. 5G).

###  Cell types were mapped to spatial cell populations and prognostic genes were demonstrated in ST sections

**Fig. 6:**
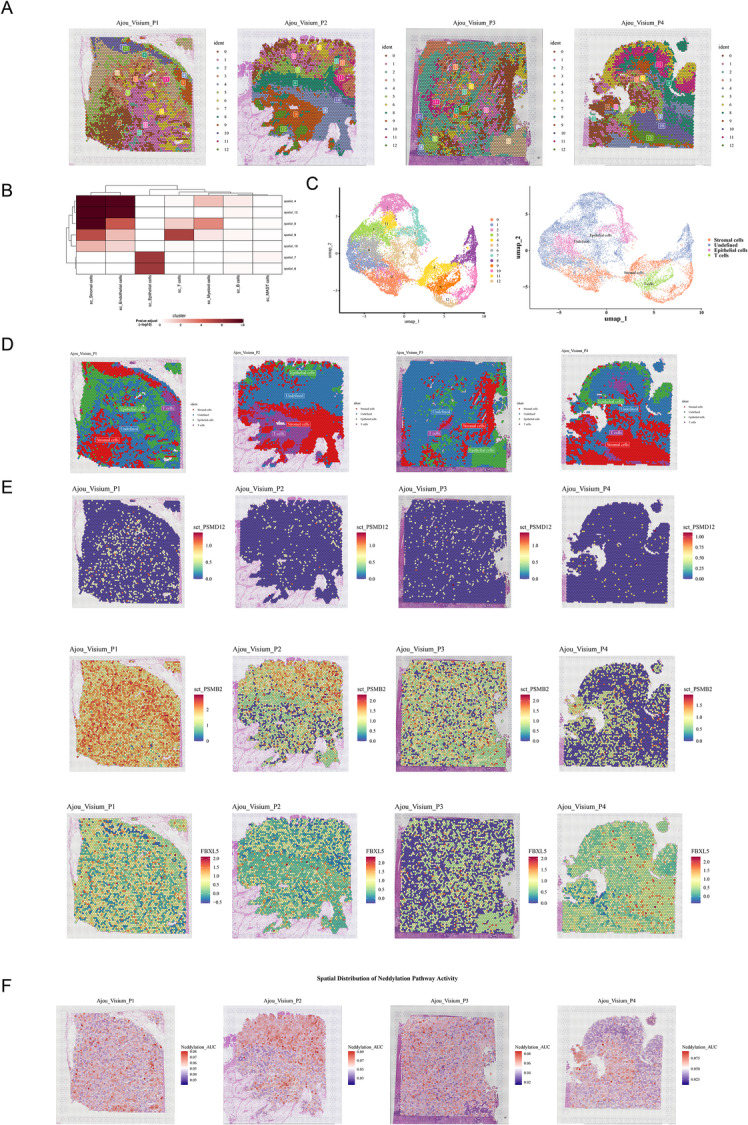
MIA and ST analysis revealing expression profiles of prognostic genes in CRC. **A** 13 cell populations of each spatial spot revealed by four patient samples (P1, P2, P3, P4) of CRC. **B** MIA of common cell populations of scRNA-seq and ST-seq. **C** Identified four cell types (epithelial cells, stromal cells, T cells and undefined cells) and generated the UMAP plots according to MIA. **D** Spatial distribution of epithelial cells, stromal cells, T cells and undefined cells in CRC samples. **E** Expression and distribution of prognostic genes (PSMD12, PSMB2, FBXL5). **F** Spatial distribution map of neddylation pathway activity

The quality of the samples from the ST dataset was initially evaluated (Figure S1). Based on the dimension value of 20, the cells were classified into 13 cell populations from 0 to 12 after dimensionality reduction clustering (Fig. 6A). A total of 4,426 cell markers were obtained among various cell types in scRNA-seq dataset. Meanwhile, 3,434 spot markers were obtained between different cell populations in the ST dataset. The above two groups of signature genes were subjected to MIA, which revealed that stromal cells exhibited a high degree of enrichment in spatial cell populations 0, 4, and 12, while endothelial cells were predominantly presented in cell populations 4 and 12, and epithelial cells were primarily distributed in cell populations 7 and 6 (Fig. 6B). A UMAP plot for the four cell types (epithelial cells, stromal cells, T cells and undefined cells) according to highest enrichment of MIA were generated (Fig. 6C). Also, the four cells were mapped to spatial cell populations (Fig. 6D). The expression distribution of prognostic genes (PSMD12, PSMB2, and FBXL5) in the sections was demonstrated and evaluated. The PSMD12 was expressed at a low level in all sections, whereas PSMB2 and FBXL5 were expressed at a slightly higher level in the sections (Fig. 6E). Finally, we assessed the spatial distribution of neddylation pathway activity. Neddylation pathway activity levels differed across the four spatial transcriptomics samples: in P1 and P3, extensive red high-activity regions were relatively abundant; in P2, red high-activity regions were primarily concentrated in the upper right quadrant; and in P4, overall activity was markedly reduced (Fig. 6F).

### Pseudo-time trajectory was constructed at epithelial cells

**Fig. 7:**
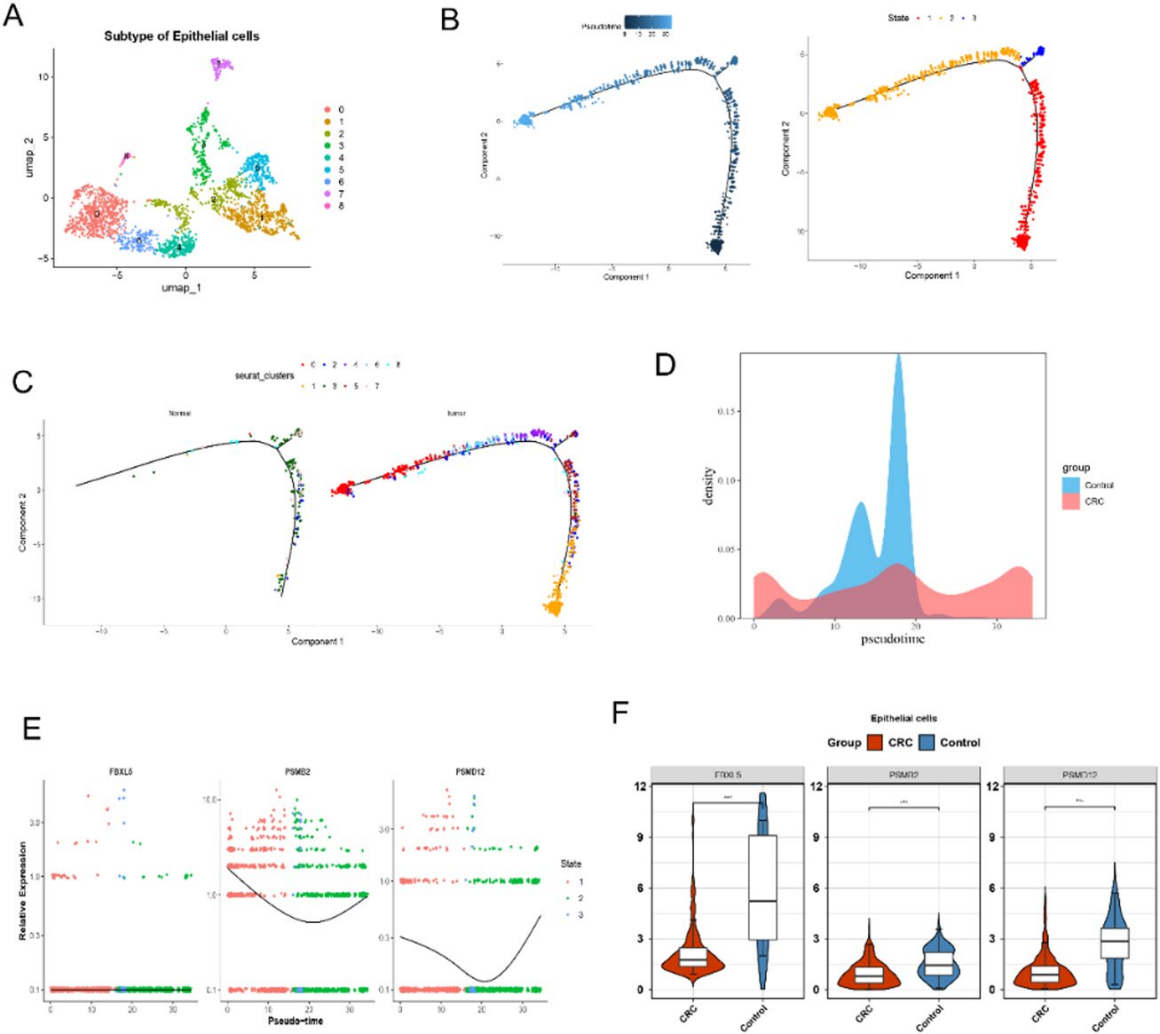
Pseudo-time temporal trajectory analysis of epithelial cells in CRC. **A** UMAP plot shows the nine subtypes of epithelial cells.** B** Pseudo-time trajectory analysis depicts the progression of epithelial cells (left) and their state transitions (right).** C** Trajectory analysis comparies normal (left) and tumor epithelial cells (right), showing distinct cellular states.** D** Density plot of pseudo-time distribution of control and CRC groups.** E** Expression of three genes (FBXL5, PSMB2, PSMB12) along the pseudo-time states.** F** Violin plots compary expression levels of three genes (FBXL5, PSMB2, PSMB12) in epithelial cells between CRC and control groups

Epithelial cells were subjected to UMAP downscaling to recluster them into nine cell subtypes (Fig. 7A). Based on known cell marker genes, these subtypes were further categorised into five major cell subpopulations: malignant cells, proliferative cells, stem-like cells, goblet cells, and enterocytes (Figture S2A-B). The pseudo-time trajectory for epithelial cells showed a total of 3 states, state 1 being predominantly cell subtype 1 in the pre-differentiation phase (Fig. 7B). Group pseudo-time trajectory analysis showed that normal epithelial cells were mostly concentrated in 1–3 states, while tumor epithelial cells were uniformly distributed (Fig. 7C). From the changes in the grouped pseudo-time trajectory density thermograms, it could be seen that the control (normal) group had a higher number of cells in the pre- and mid-stage, which eventually transformed to tumor cells (Fig. 7D). Neddylation activity also exhibited distinct patterns during differentiation. Pathway activity was elevated in certain cells during both early and late differentiation stages, whereas overall activity tended to be lower in the intermediate branch region (Figture S2C). The demonstration of the changes in the three prognostic genes in the pseudo-time trajectory analysis revealed a trend of initial decrease and subsequent increase in the expression of both PSMD12 and PSMB2 in different developmental stages of epithelial cells (Fig. 7E). Furthermore, their expression levels were higher in cells within regions of high neddylation activity (Figture S2D). In contrast, FBXL5 demonstrated lower expression across these stages. Furthermore, the prognostic genes were significantly down-expressed in CRC group relative to controls (Fig. 7F).

### The lncRNA-miRNA-mRNA and drugs networks were constructed with prognostic genes

**Fig. 8:**
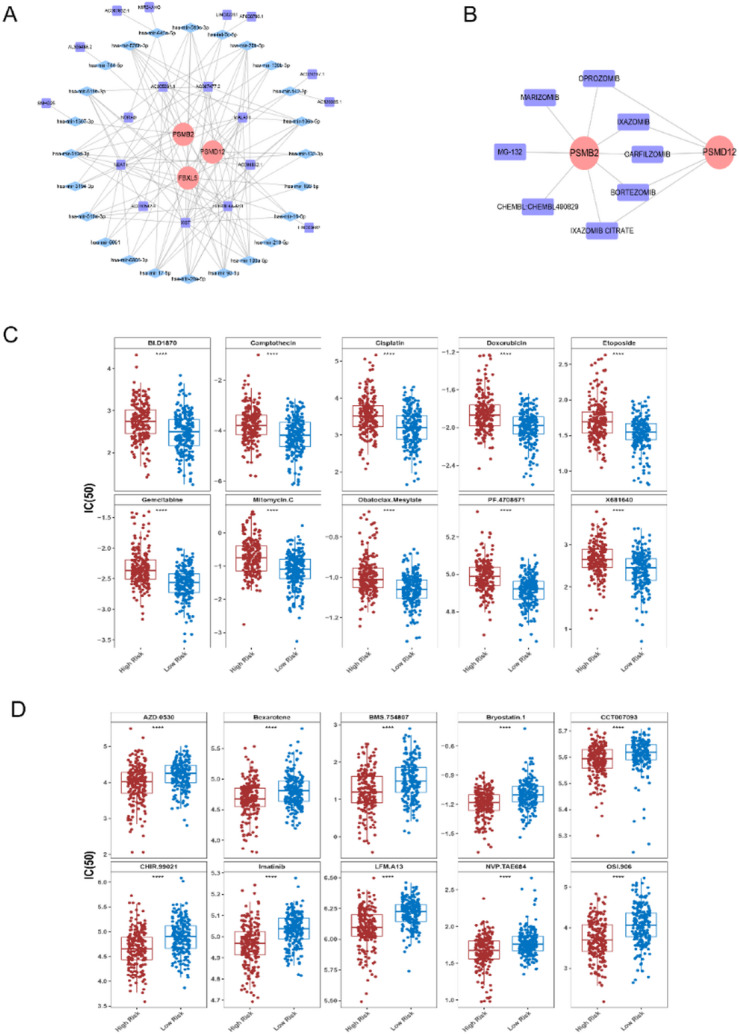
Prognostic gene lncRNA-miRNA-mRNA network prediction and drug sensitivity analysis. **A** LncRNA-miRNA-mRNA network of prognostic gene (red: mRNA, blue: miRNA, purple: lncRNA).** B** Potential drugs-prognostic genes network, (Red: target, purple: drug).** C**,** D** Boxplots of IC50 values for top 10 drugs lower sensitivity (**C**) and top 10 drug high sensitivity (**D**) of high-risk groups compared with low-risk groups (red: high-risk, blue: low-risk groups)

For three prognostic genes, 30 miRNAs were predicted against PSMD12, 47 miRNAs against PSMB2, and six miRNAs against FBXL5, including a total of 83 non-repeat key miRNAs. Further, 18 lncRNAs were predicted and obtained based on 24 key miRNAs. Thus, the lncRNA-miRNA-mRNA network was displayed to elucidate their regulatory relationship (Fig. 8A). A search for potential drugs or molecular compounds related to prognostic targets through the DGIdb database yielded 8 drugs, of which 8 were retrieved by PSMB2, 5 by PSMD12 (Fig. 8B). Among them, bortezomib, oprozomib, carfilzomib, ixazomib citrate, and ixazomib were searched together for both genes. Inter high- and low-risk cohorts, 51 drugs had significantly higher IC_50_ values in high-risk cohort than in low-risk cohort, for example, camptothecin, cisplatin, and doxorubicin (Fig. 8C). Meanwhile, 29 drugs had significantly lower IC_50_ values in high-risk cohort than in the low-risk cohort, for example, bexarotene, bryostatin.1, and imatinib (Fig. 8D).

## Discussion

CRC ranks as the third most common and second most lethal cancer globally, with rising incidence rates in both developed and middle- to low-income countries, driven by westernization [27]. Neddylation is a critical regulatory mechanism in the malignant transformation of tumor cells, including CRC. It affects the stability and function of key proteins such as HuR and SHP2, which influence both tumor cell behavior and the tumor microenvironment’s interaction with the immune system [28, 29]. Abnormal neddylation affects the ubiquitin-proteasome system, leading to increased stability of oncogenic proteins and disrupting normal cell cycle regulation in CRC epithelial cells [30]. A study has indicated that targeting the neddylation pathway in intestinal epithelial cells inhibits NF-κB, triggering persistent apoptosis [31]. Thorough investigation of the role of neddylation in CRC and its regulatory mechanisms is of great significance for the discovery of new therapeutic targets and the development of personalized treatment approaches.

This study employed an integrative analysis of scRNA-seq, ST-seq and transcriptomics to identify candidate genes involved in the neddylation process in CRC. By intersecting 5,131 DEGs(sc), 9,089 DEGs(bulk), and 247 NRGs, 32 candidate genes were obtained. Univariate Cox, LASSO Cox, and multivariate Cox regression analysis identified the prognostic significance of neddylation-related genes including PSMD12, PSMB2, and FBXL5. In addition, a lncRNA-miRNA-mRNA network and a drug-prognostic gene network were constructed, leading to the identification of some potential drugs like bortezomib.

scRNA-seq and ST-seq have provided a comprehensive map of neddylation-related gene expression at both the cellular and spatial levels in CRC. We identified seven distinct cell populations, including T cells, B cells, epithelial cells, myeloid cells, stromal cells, mast cells, and endothelial cells. Among these, FBXL5 and PSMB2 exhibited increased expression in CRC epithelial cells when compared to other cell types. However, in contrast to normal epithelial tissues, the expression of FBXL5, PSMB2, and PSMD12 was markedly decreased in tumor epithelial cells.

Furthermore, through risk score analysis based on bulk-seq, we observed that the elevated expression of FBXL5, PSMB2, and PSMD12 was inversely correlated with poor prognosis, suggesting their protective role in CRC. Importantly, risk scores derived from these prognostic genes, along with age, T- and N-stages, were identified as independent predictors of CRC.

PSMD12 (Proteasome 26 S Subunit, Non-ATPase 12, 26 S proteasome non-ATPase regulatory subunit 12), This complex is crucial for maintaining protein homeostasis by facilitating the removal of misfolded or damaged proteins that could disrupt cellular function [32]. Studies have found that PSMD12 promotes the development of liver cancer by upregulating KIF15 and promoting the activation of the MEK-ERK pathway [33]. Additionally, PSMD12 interacts with CDKN3 and facilitates the progression of pancreatic cancer [34]. However, Amino Acid Metabolism Reprogramming found PSMD12 as a protective factor in colon cancer [35], aligning with our findings. Therefore, it is hypothesized that PSMD12 has potential as a key target for the treatment of CRC.

PSMB2, the non-catalytic subunit of the 20 S core proteasome complex is integral to the proteolytic degradation of the majority of intracellular proteins. This complex is crucial for various cellular functions, as it interacts with multiple regulatory particles [36]. Bioinformatics analysis identified PSMB 1/2/3/4/6/8/9/10 as a prognostic marker for clear cell renal cell carcinoma [37]. PSMB2 also acts as an oncogene in glioma and is associated with the immune microenvironment [38]. Research has been conducted on PSMB1 as a biomarker for CRC. The study identified PSMB1 as a potential biomarker and therapeutic target for CRC. Kinetin, an anticancer drug, enhances the degradation of proteasome-dependent oncoprotein [39]. The findings of this study provide further evidence that the PSMB1 is a potential therapeutic target for CRC and warrants further investigation in the future.

Our study results demonstrated that the proteasome subunits PSMD12 and PSMB2 are downregulated in high-risk populations of CRC. Decreased expression levels of these two subunits may lead to a subsequent reduction in the protein degradation efficiency of the proteasome, which in turn may result in the stable accumulation of oncogenic proteins and promote cancer progression. Additionally, previous studies have revealed a negative feedback regulatory mechanism of the proteasome on upstream ubiquitinating enzymes. Specifically, appropriate proteasome function can facilitate the deneddylation modification of Cullin proteins, thereby playing a key role in regulating the recycling and reactivation of Cullin-RING Ligases (CRLs) [40]. Based on this, we hypothesize that downregulation of PSMD12 and PSMB2 may induce proteasome dysfunction, which could disrupt this negative feedback mechanism. Such disruption may reduce the efficiency of Cullin protein deneddylation, leading to their abnormal sustained activation through neddylation. This persistent activated state may trigger hyperactivity of CRL E3 ligases, resulting in massive intracellular accumulation of toxic ubiquitinated proteins and severe impairment of protein homeostasis [41]. Collectively, these events contribute to tumor initiation and progression. However, this mechanism remains speculative. Future studies should utilize cell models to knockdown PSMD12/PSMB2, and observe the effects on Cullin protein neddylation levels, CRL activity, and cellular malignant phenotypes, thereby verifying the specific role of the aforementioned molecular pathway in colorectal cancer progression.

Our study identified FBXL5 as a neddylation-related prognostic gene in CRC. Neddylation modification can regulate the stability of substrate proteins by activating Cullin-RING E3 ubiquitin ligases [42]. FBXL5 itself is a key component responsible for substrate recognition in the SCF E3 ubiquitin ligase complex [43], and its function may be directly regulated by the neddylation pathway. Additionally, studies have indicated that FBXL5 and its substrate IREB2 are regarded as Cullin-associated proteins undergoing neddylation modification [44]. FBXL5 plays a central role in maintaining cellular iron homeostasis by promoting the ubiquitin-mediated and subsequent proteasomal degradation of IRP2 [45, 46]. FBXL5 contains a [2Fe_2_S] cluster that is essential for the recruitment of IRP2 recruitment, and they govern the stability of iron levels within the cell [47]. FBXL5 is also vital in mediating cellular ferroptosis [48]. In our study, downregulated FBXL5 expression in the high-risk group suggests impaired function, which may reduce the ability of the neddylation pathway to regulate iron homeostasis through FBXL5. This loss of function might inhibit ferroptosis in tumor cells, ultimately being closely associated with poor prognosis. Furthermore, studies have revealed that FBXL5 acts as an oncogene in CRC progression by regulating the PTEN/PI3K/AKT signaling pathway in CRC tissues and cell lines [49]. Therefore, FBXL5 exhibits significant value in CRC treatment through its critical roles in activating ferroptosis, neddylation, and oncogenic signaling. However, the interaction between FBXL5-mediated iron homeostasis regulation and oncogenic signaling pathways in the tumor microenvironment, as well as how they are coordinated by the global regulatory network of neddylation, requires further experimental investigation.

The lncRNA-miRNA-mRNA network predicted in this study may play regulatory mechanisms in CRC, which has been confirmed by many studies. Among them, hsa-miR-106b-5p has been found to act as a tumor suppressor factor in CRC. Its low expression linked to the enhanced invasion and metastasis ability of cancer cells [50]. In addition, through its competitively binding to miR-106b-5p, MALAT1 regulates the microtubule activity of SLAIN2, thereby promoting the progression and poor prognosis of CRC [51]. In terms of lncRNAs, NEAT1 and MALAT1 have been extensively studied. NEAT1 competitively binds to miR-34a, inhibits SIRT1 and activates the Wnt/β-catenin signaling pathway, thereby promoting the proliferation and metastasis of CRC [52]. In addition, NEAT1 also interacts with miR-195-5p in the ceRNA network, affecting the immune infiltration and patient prognosis of CRC [53]. Therefore, further exploration of miRNAs and lncRNAs is significant implications for understanding the pathogenesis and treatment of CRC.

Analysis of drug sensitivity revealed notable differences in how high- and low-risk groups responded to particular chemotherapeutic agents., offering potential therapeutic drugs for personalized medicine. The high-risk group demonstrated lower IC50 values and higher sensitivity to certain drugs such as Bexarotene and Imatinib, while showing high IC50 values and low sensitivity to Camptothecin, Cisplatin, Doxorubicin, and Gemcitabine. The high-risk group shows limited sensitivity to commonly used chemotherapeutic drugs for CRC, but may be potentially adaptable to some drugs in the trial stage. Imatinib has demonstrated synergistic pleiotropic effects in inhibiting pro-inflammatory, cell survival, and angiogenesis signals in the prevention of CRC [54]. Studies have indicated that the combination of imatinib with different drugs, such as bevacizumab, can suppress CRC cells growth and is both safe and well-tolerated free from notable drug interactions [55]. The pharmacokinetics (PK) of imatinib in combination with Oxaliplatin and 5FU in the treatment of CRC patients remains unchanged [56].

Previous works predominantly focuses on the overarching function of protein neddylation in human cancers and potential therapeutic strategies [57, 58]. In contrast, this study specifically zeroed in on CRC and integrated the single-cell and spatial transcriptomics was employed to identify prognostic genes associated with neddylation in CRC for a novel perspective. Our results are not only align with previous studies [29, 59]review on neddylation’s implications in CRC, but also specifically identifies PSMD12, PSMB2, and FBXL5 as potential prognostic genes and an effective prognostic model. This prognostic model facilitates the stratification of patients into distinct risk groups, enabling personalized treatment strategies and more precise clinical management [60]. This model’s predictive capacity is bolstered by its ability to identify specific molecular targets, thereby guiding the selection of targeted therapies [61]. Although this study represents a modest advancement in the field, it is important to acknowledge the limitations of our work. This study primarily identified prognostic genes based on bioinformatics analyses, and the expression of these prognostic genes as well as their specific functions in CRC have not yet been validated in independent samples through experiments such as quantitative polymerase chain reaction (qPCR), immunohistochemistry (IHC), or functional assays. Therefore, the current work focuses on the identification of prognostic genes, with insufficient exploration of the underlying molecular mechanisms. Future studies should extend our work by first validating the expression and functions of core genes experimentally, followed by in-depth investigation of the complex mechanisms through which neddylation and these genes act in CRC, thereby providing insights for the development of targeted therapies.

## Materials and methods

### Data source

The Cancer Genome Atlas (TCGA)-CRC was drafted from TCGA database (http://cancergenome.nih.gov/), which was accessed on 6 August 2024. TCGA-CRC dataset included RNA sequencing data, clinical information, and survival information from 606 CRC tumour tissue samples and 51 normal tissue samples. Of which, 597 CRC samples with > 30 days of survival were sorted into a training set and an internal validation set by 7: 3. The external validation set, single-cell sequencing (scRNA-seq) dataset, and spatial transcriptome (ST) dataset were all downloaded from the Gene Expression Omnibus (GEO) website (https://www.ncbi.nlm.nih.gov/geo/). GSE28722 (GPL13425, external validation set) had 125 CRC tumour tissue samples containing survival information. GSE132257 (GPL16791, scRNA-seq dataset) contained 5 CRC tissue samples and 5 paracancerous normal colon tissue samples. GSE226997 (GPL30173, ST dataset) contained data on gene expression profiles, tissue sections, and spatial location information from 4 CRC tissue samples. In addition, 247 neddylation related genes (NRGs) were generated by searching ‘Neddylation’ on the Reactome database (https://reactome.org/) (Table S1).

### Analysis for scRNA-seq data

The single-cell data were read using CreateSeuratObject function of “Seurat” package (version 1.36.3) [62] and filtered to exclude cells with nFeature > 5,000, nCount > 4,000, and mitochondrial genes greater than 10%. After further evaluation of the data, the FindVariableFeatures function was then applied to get highly variable genes between cells, with the screening parameters selection.method = vst and nfeatures = 2,000. The highly variable genes from the above screen were normalized using the ScaleData function and then subjected to principal component analysis (PCA). A linear dimensionality reduction was processed using JackStraw and ScoreJackStraw functions to gain principal components (PCs). A resolution of 0.4 was set for dimensionality reduction clustering, and cell clusters were obtained by dimensionality reduction analysis of the samples based on the above PCs using the Uniform Manifold Approximation and Projection (UMAP) algorithm to annotate cell types. Cell subpopulations were annotated and visualized using marker genes from reference [63] to identify different cell types. With the aim of exploring the main biological functions of each cell type in the scRNA-seq data, all samples were subjected to gene set variation analysis (GSVA) by “ReactomeGSA” package (version 1.12.0) [64] to explore their biological pathways. Simultaneously, cell communication was analyzed for cell types using the “CellChat” package (version 1.6.1) [65]. Moreover, differential analysis of different cell types between CRC and control groups was performed by FindMarkers function, and only genes with fold change (FC) greater than 1.2-fold were retained in the results. And the screening threshold for differentially expressed genes (DEGs) was average |average log_2_FC| > 0.25 and *p* value < 0.05. The cell type with the most DEGs(sc) was deemed as key cell for further analysis.

### Screening for candidate genes

The “DESeq2” package (version 1.36.0) [66] was implemented to compare the differences in gene expression levels between CRC and control samples in TCGA-CRC dataset, with p adj. < 0.05, and |log_2_FC| > 0.5, to produce DEGs(bulk). Adjusted p value was achieved by controlling for the false discovery rate (FDR). A volcano plot was drawn using “ggplot2” package (version 3.3.0) [67] to show DEGs(sc), and the top 3 up-regulated and down-regulated genes were labeled. A heatmap was performed using “pheatmap” package (version 0.7.7) [68], which showed the expression of top 10 DEGs(bulk) with positive and negative log_2_FC. Intersections of DEGs in DEGs(sc)(key cells), DEGs(bulk), and NRGs were taken to yield candidate genes that were linked to neddylation and differed in both the single-cell transcriptome and the bulk transcriptome. Furthermore, Gene Ontology (GO) and the Kyoto Encyclopedia of Genes and Genomes (KEGG) were analyzed with *p* < 0.05 for probing possible roles of candidate genes with the aid of “clusterProfiler” package (version 3.8.1) [69]. The Search Tool for Recurring Instances of Neighbouring Genes (STRING) database (https://string-db.org/) was then manipulated to construct a protein-protein-interaction (PPI) network with a confidence level of 0.4.

### Construction of prognostic model

To ensure the reliability of prognostic genes and prevent the occurrence of over fitting phenomenon, this study set up a training set, an internal validation set and an external validation set. Candidate genes were first subjected to a univariate Cox analysis (*p* < 0.05) and proportional hazards (PH) assumption test (*p* > 0.05) in training set, which were used to produce genes that were associated prominently with prognosis. For reducing the feature dimension, the prognostic associated genes gotten by univariate Cox and PH test were inputted into least absolute shrinkage and selection operator (LASSO) by “glmnet” package (version 4.1-4.1) [70] with family setting as Cox, and the strongly correlated features were selected to generate prognostic genes when lambda was taken to its minimum value and coefficients for a risk model.

$$\:risk\:score=\sum\:_{n=1}^{n}(\text{c}\text{o}\text{e}\text{f}\text{f}\text{i}\text{c}\text{i}\text{e}\text{n}\text{t}\:i\times\:expression\:i)$$

In the formula, n stands for the number of genes, *i* stands for prognostic genes. The prognostic risk model was evaluated and validated in three datasets. The risk score was calculated with prognostic risk model, and the optimal threshold was chosen with the surv_cutpoint function, which was applied for dividing into high-/low-risk cohorts for CRC samples. Risk score curves and patient prognosis were presented in each dataset. The prognostic model was then assayed by receiver operating characteristic (ROC), as well as Kaplan–Meier (K–M) curves between two cohorts (*p* < 0.05). Also, expression of prognostic genes in each dataset was demonstrated with heatmaps.

### Independent prognostic analysis

To assess the capacity of risk scores and clinical factors (age, gender, race, T and N stages) as independent prognostic factors, a comprehensive suite of analytical tools was employed, entailing the execution of univariate Cox with *p* < 0.05, PH test with *p* > 0.05, and multivariate Cox analyses with *p* < 0.05, utilising the “survival” package (version 3.4-0.4) [71]. Then, nomogram model was developed with independent prognostic factors by “rms” package (version 5.1-4.1) [72]. Accuracy for nomogram was assessed by calibration and ROC curves. Additionally, differences of risk scores in different clinical factors were analyzed by Wilcoxon (two groups) or kruaskal test (multiple groups) (*p* < 0.05).

### Function pathways of high- and low-risk cohorts

Differential analysis was implemented on two risk cohorts from training set by means of “Deseq2” package (version 1.36.0), and genes were sorted according to differential expression folds, followed by gene set enrichment analysis (GSEA) using “clusterProfiler” package (version 3.8.1) with *p* < 0.05. Reference gene set, c2.cp.kegg.v2023.1.Hs.symbols.gmt, was collected from the Molecular Signatures Database (MSigDB, https://www.gsea-msigdb.org/gsea/msigdb/). The |normalized enrichment score (NES)| > 1 and *p* < 0.05 were used as thresholds for pathways, and the top 3 pathways with positive or negative enrichment scores were selected for figure mapping.

### Analysis of ST samples

In order to examine the diverse spatial distributions of ST data, the data were subjected to statistical analysis using “Seurat” package (version 1.36.3). The SpatialFeaturePlot function was employed to visualize the total number of genes (nFeature) and sum of gene expression (nCount) of the samples within the ST dataset. Next, the ST data of the samples were normalized using the SCTransform function, and linear dimensionality reduction was used to obtain the linear optimal dimensionality value of the cell populations, and based on the dimensionality value, the subsequent cell cell populations were classified and plotted in an UMAP diagram for display.

### Multimodal intersection analysis (MIA)

In order to understand spatial distribution of cell populations, FindAllMarkers function was first used to screen for signature genes (cell markers) among various cell types in scRNA-seq dataset based on average log_2_FC > 0.3 and p adj. < 0.05 by FDR. Additionally, this was also done for signature genes (spot markers) among different cell populations in the ST dataset according to same threshold. Subsequently, enrichment scores (ES) were calculated by quantifying the enrichment levels of characteristic gene sets between spatial transcriptomic clusters and single-cell transcriptomic cell types using MIA. Based on the ranking of enrichment scores, each spatial cluster was annotated with the cell type exhibiting the highest ES value, thereby elucidating its spatial distribution pattern. Besides, expression distribution of prognostic genes was demonstrated in all sections. Finally, to investigate the spatial regulatory characteristics of the neddylation pathway, we calculated neddylation pathway activity scores (AUC values) for four spatial transcriptomics samples using the AUCell package (version 1.20.1) [73], based on its associated gene sets. Subsequently, the calculated AUC scores were integrated into spatial coordinate data, and the spatial distribution of neddylation pathway activity for each sample was plotted using the SpatialFeaturePlot function from the Seurat package.

### Pseudo-time analysis

To construct a single cell trajectory map, pseudo-time analysis was undertaken for key cells. The key cells were first subjected to UMAP downscaling to recluster into different molecular subtypes. Subsequently, cell subpopulations were annotated and visualised based on marker genes from the reference to identify different cell types [10]. Finally, pseudo-time analysis of different subtypes within key cells was analyzed using the “Monocle” package (version 2.18.0) [74]. Furthermore, to investigate pathway activity changes during development, AUCell R package (version 1.20.1) was employed to assess activity scores of Neddylation-related gene sets in epithelial cells. These scores were integrated into the constructed pseudo-time trajectories and visualised using the “Monocle” package.

### Molecular regulatory network and drug-related analyses

With the purpose of investigating the potential regulatory mechanisms of prognostic genes in CRC, the miRTarBase on NetworkAnalyst database (https://www.networkanalyst.ca/NetworkAnalyst/) was first employed to anticipate miRNAs corresponding to each prognostic gene. The key miRNAs were obtained by removing duplicates from the above miRNAs. Subsequently, the lncRNAs corresponding to these key miRNAs were gotten by Starbase database (http://starbase.sysu.edu.cn/) with clipExpNum ≥ 10. Ultimately, the aforementioned outcomes were synthesized to develop a mRNA-miRNA-lncRNA network. To further assess the association inter two risk cohorts and response to chemotherapy, “pRRophetic” (version 0.5) [75] was deployed to calculate the half-maximal inhibitory concentrations (IC_50_) values of 138 common drugs for all CRC patients in the TCGA-CRC dataset. The top 10 drugs with the most significant differences between risk cohorts were shown using “ggplot2” package (version 3.3.0). In addition, potential drug or molecular compounds related to prognostic genes were derived through Drug-Gene Interaction database (DGIdb, https://www.dgidb.org/).

### Statistical analysis

The R software, version 4.2.2, was employed for the purpose of performing statistical analyses. In order to compare the differences between two groups, the Wilcoxon test was utilized, with a p value of less than 0.05 being considered statistically significant.

## Conclusions

This study comprehensively explored the role of neddylation in colorectal cancer (CRC) using single-cell RNA sequencing (scRNA-seq), spatial transcriptomics (ST), and bulk transcriptome data. We identified 32 candidate genes related to neddylation and CRC, among which PSMD12, PSMB2, and FBXL5 were found to be prognostic genes. The prognostic risk model based on these genes demonstrated good predictive accuracy, with low expression of the three genes associated with high risk and poor prognosis. The study also revealed the spatial distribution of cell types and the expression patterns of prognostic genes in CRC, highlighting their potential as therapeutic targets. Additionally, the construction of lncRNA-miRNA-mRNA and drug-prognostic gene networks provided further insights into the regulatory mechanisms and potential drugs for CRC treatment.

## Supplementary Information

Supplementary Material 1: Figure S1: The quality of the samples from the CRC ST dataset; Table S1: The list of NRGs; Table S2: PH test for 32 candidate genes; Table S3: PH test of risk score and clinical factors.

## Data Availability

Data Availability Statement: The TCGA-CRC dataset, including RNA sequencing, clinical, and survival data from 606 CRC tumor and 51 normal samples, was accessed from the TCGA database (http://cancergenome.nih.gov/) on 6 August 2024. Data were split into a 7:3 training and internal validation set. The external validation set, scRNA-seq, and ST datasets were obtained from GEO (https://www.ncbi.nlm.nih.gov/geo/): GSE28722 (GPL13425) includes 125 CRC tumor samples with survival data, GSE132257 (GPL16791) contains scRNA-seq data from 5 CRC and 5 normal samples, and GSE226997 (GPL30173) provides ST data from 4 CRC samples. Neddylation-related genes were identified from the Reactome database (https://reactome.org/). All datasets generated in this study are included within the article and its supplementary materials.

## Abbreviations

CRC: Colorectal cancer
NRGs: Neddylation-related genes
DEGs: Differentially expressed genes
scRNA-seq: Single-cell RNA sequencing
ST: Spatial transcriptome
TCGA: The Cancer Genome Atlas
GEO: Gene expression omnibus
GSE: Gene expression omnibus series
GPL: Gene expression omnibus platform
GSM: Gene expression omnibus sample
PTM: Post-translational modification
Nedd8: Neural precursor cell expressed, developmentally down-regulated 8
ST: Spatial transcriptomics
GSVA: Gene set variation analysis
PCA: Principal component analysis
UMAP: Uniform Manifold Approximation and Projection
GO: Gene ontology
KEGG: Kyoto Encyclopedia of Genes and Genomes
PPI: Protein-protein interaction
LASSO: Least absolute shrinkage and selection operator
ROC: Receiver operating characteristic
K–M: Kaplan–Meier
AUC: Area under the curve
GSEA: Gene set enrichment analysis
MIA: Multimodal intersection analysis
lncRNA: Long non-coding RNA
miRNA: MicroRNA
mRNA: Messenger RNA
IC50: Half-maximal inhibitory concentrations
DGIdb: Drug-gene interaction database
MSigDB: Molecular signatures database
