# Supplementary material for: Integration analysis of single-cell and spatial transcriptomics identifies prognostic genes associated with neddylation in colorectal cancer
Source: Discov Oncol. 2025 Dec 24;16:2224. doi: 10.1007/s12672-025-04340-y (PMC12748437; doi:10.1007/s12672-025-04340-y)
Supplement: Supplementary file 1 — Supplementary Material 1: Figure S1: The quality of the samples from the CRC ST dataset; Table S1: The list of NRGs; Table S2: PH test for 32 candidate genes; Table S3: PH test of risk score and clinical factors. [file 12672_2025_4340_MOESM1_ESM.docx]

**Supplement**

**Figture S1.** The quality of the samples from the CRC ST dataset


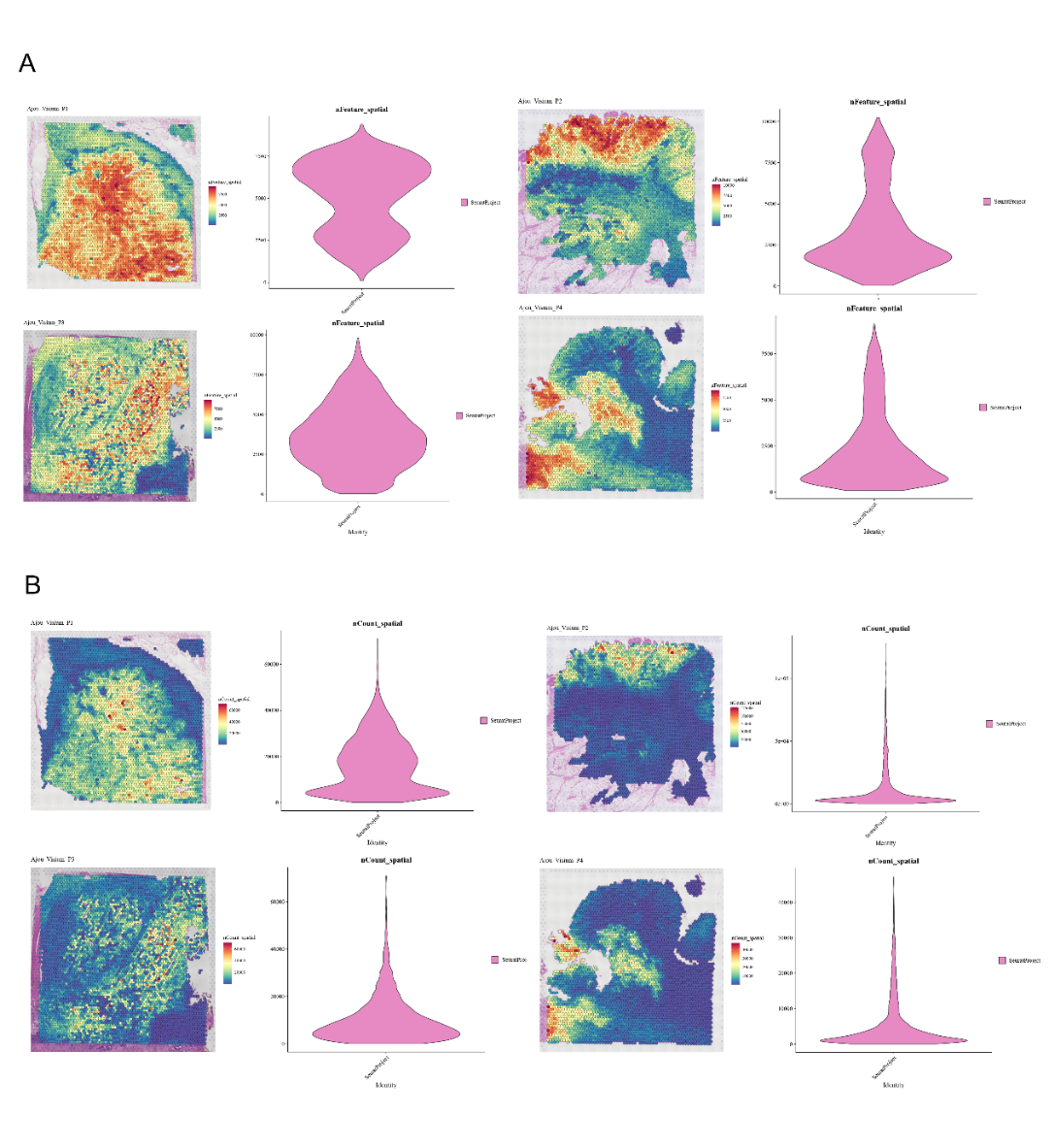


**Figture S1.** The quality of the CRC samples from the ST dataset. (A) Spatial feature plots (left) and violin plots (right) show nFeature_RNA of spatial distribution four CRC tissue sections samples. (B) Spatial feature plots (left) and violin plots (right) show nCount_RNA of spatial distribution four CRC tissue sections samples. Color scales represent expression or count levels from low (blue) to high (red).


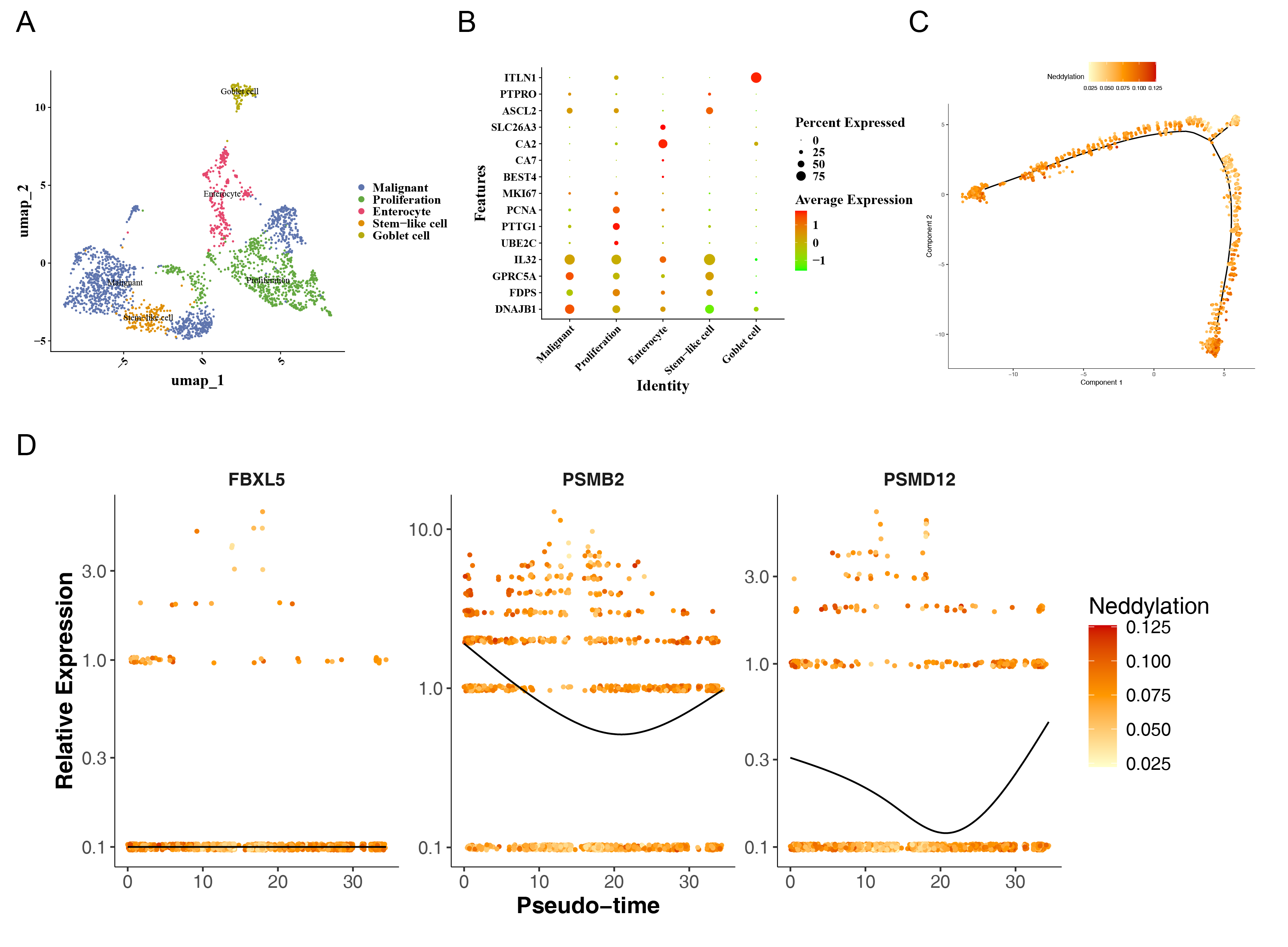


**Figture S2** Subpopulation analysis of epithelial cells and neddylation pathway activity analysis. (A) UMAP plot of distinct epithelial cell subpopulations. (B) Marker gene expression across different cell types. (C) Neddylation pathway activity in epithelial cells along pseudo-time trajectories. (D) PSMD12 and PSMB2 expression in epithelial cells may correlate with neddylation activity.

***Table S1.*** *The list of NRGs*

| **Numbers** | **NRGs** |
| --- | --- |
| 1 | KLHL13 |
| 2 | FBXL3 |
| 3 | ASB4 |
| 4 | PSMB1 |
| 5 | BRCA1 |
| 6 | PSMC4 |
| 7 | NUB1 |
| 8 | CUL3 |
| 9 | PSMA4 |
| 10 | DCUN1D1 |
| 11 | CUL7 |
| 12 | ERCC8 |
| 13 | CUL1 |
| 14 | PUM2 |
| 15 | BTBD1 |
| 16 | ASB1 |
| 17 | PSME4 |
| 18 | UFD1 |
| 19 | UBE2D1 |
| 20 | FBXW11 |
| 21 | KLHL20 |
| 22 | KEAP1 |
| 23 | PALB2 |
| 24 | PSMC5 |
| 25 | KLHL42 |
| 26 | BIRC5 |
| 27 | MUL1 |
| 28 | PSME1 |
| 29 | PSMD5 |
| 30 | PSMD8 |
| 31 | FBXL19 |
| 32 | KLHL22 |
| 33 | FBXO7 |
| 34 | RBX1 |
| 35 | PSMC6 |
| 36 | PSMA3 |
| 37 | ASB2 |
| 38 | HIF1A |
| 39 | PSMC1 |
| 40 | PSMB5 |
| 41 | DCAF11 |
| 42 | PSMA6 |
| 43 | PSME2 |
| 44 | PSMA7 |
| 45 | PSMD10 |
| 46 | CCDC22 |
| 47 | ASB9 |
| 48 | RBBP7 |
| 49 | PSMD7 |
| 50 | FBXO31 |
| 51 | ELOB |
| 52 | PSMA2 |
| 53 | FBXW4 |
| 54 | FBXL15 |
| 55 | CUL2 |
| 56 | FBXL20 |
| 57 | PSMD3 |
| 58 | PSMD11 |
| 59 | WSB1 |
| 60 | DCUN1D4 |
| 61 | UBE2D3 |
| 62 | KLHL2 |
| 63 | FBXW7 |
| 64 | KLHL5 |
| 65 | ZBTB16 |
| 66 | COMMD9 |
| 67 | PSMD9 |
| 68 | CAND1 |
| 69 | COPS7A |
| 70 | SPSB2 |
| 71 | LOC105369632 |
| 72 | FBXO9 |
| 73 | FBXL4 |
| 74 | CUL9 |
| 75 | SKP1 |
| 76 | RNF7 |
| 77 | CISH |
| 78 | COMMD2 |
| 79 | PSMD14 |
| 80 | ASB3 |
| 81 | GPR75-ASB3 |
| 82 | DCAF17 |
| 83 | EPAS1 |
| 84 | NFE2L2 |
| 85 | FBXO2 |
| 86 | FBXO6 |
| 87 | RBBP5 |
| 88 | FBXO30 |
| 89 | FBXL5 |
| 90 | UCHL3 |
| 91 | FBXW2 |
| 92 | DCAF4 |
| 93 | KBTBD7 |
| 94 | SOCS2 |
| 95 | COPS5 |
| 96 | DCAF10 |
| 97 | OBSL1 |
| 98 | NEURL2 |
| 99 | HIF3A |
| 100 | CDKN1A |
| 101 | PSMF1 |
| 102 | PSMB2 |
| 103 | FBXL12 |
| 104 | FBXL16 |
| 105 | SEM1 |
| 106 | PSMA1 |
| 107 | NEDD8 |
| 108 | DDA1 |
| 109 | TULP4 |
| 110 | UBE2M |
| 111 | PSME3 |
| 112 | UBE2D2 |
| 113 | FBXW9 |
| 114 | LRRC41 |
| 115 | DCAF8 |
| 116 | FBXO44 |
| 117 | VHL |
| 118 | DDB2 |
| 119 | FBXO21 |
| 120 | FBXL8 |
| 121 | LMO7 |
| 122 | DCAF7 |
| 123 | PSMB7 |
| 124 | DCUN1D5 |
| 125 | FBXO11 |
| 126 | COPS4 |
| 127 | CUL4A |
| 128 | DCAF5 |
| 129 | COMMD4 |
| 130 | COPS3 |
| 131 | FBXO15 |
| 132 | FEM1A |
| 133 | PSMB6 |
| 134 | WDTC1 |
| 135 | PSMA5 |
| 136 | DCAF6 |
| 137 | COP1 |
| 138 | DTL |
| 139 | RPS27A |
| 140 | COPS7B |
| 141 | UBA3 |
| 142 | SKP2 |
| 143 | FEM1C |
| 144 | COMMD10 |
| 145 | KLHL3 |
| 146 | ASB15 |
| 147 | ASB10 |
| 148 | FBXO10 |
| 149 | ASB6 |
| 150 | COMMD3 |
| 151 | COMMD7 |
| 152 | DCUN1D2 |
| 153 | UBC |
| 154 | FBXO4 |
| 155 | ASB17 |
| 156 | ELOC |
| 157 | PSMA8 |
| 158 | FBXL18 |
| 159 | ANKRD9 |
| 160 | FBXO32 |
| 161 | CUL4B |
| 162 | FBXW5 |
| 163 | PSMD4 |
| 164 | PSMB4 |
| 165 | NAE1 |
| 166 | SQSTM1 |
| 167 | FBXL13 |
| 168 | PSMC2 |
| 169 | FBXO27 |
| 170 | ASB16 |
| 171 | SPSB3 |
| 172 | CCNF |
| 173 | KLHL21 |
| 174 | FBXO41 |
| 175 | DCAF16 |
| 176 | KBTBD8 |
| 177 | PSMD6 |
| 178 | FBXO40 |
| 179 | UBXN7 |
| 180 | FBXW12 |
| 181 | ASB5 |
| 182 | DCAF13 |
| 183 | ASB11 |
| 184 | VCP |
| 185 | LRR1 |
| 186 | KBTBD6 |
| 187 | PSMC3 |
| 188 | BTRC |
| 189 | SENP8 |
| 190 | COPS2 |
| 191 | CUL5 |
| 192 | FBXO22 |
| 193 | DDB1 |
| 194 | COPS6 |
| 195 | KCTD6 |
| 196 | FEM1B |
| 197 | COMMD8 |
| 198 | CCDC8 |
| 199 | GPS1 |
| 200 | UBB |
| 201 | COMMD5 |
| 202 | SOCS6 |
| 203 | SOCS5 |
| 204 | SPSB1 |
| 205 | FBXL14 |
| 206 | FBXW10 |
| 207 | COMMD1 |
| 208 | PSMD1 |
| 209 | FBXW8 |
| 210 | SPSB4 |
| 211 | PSMD2 |
| 212 | WSB2 |
| 213 | ASB8 |
| 214 | KLHL11 |
| 215 | ASB18 |
| 216 | NPLOC4 |
| 217 | ASB7 |
| 218 | FBXL7 |
| 219 | KLHL25 |
| 220 | UBE2F |
| 221 | SOCS3 |
| 222 | AMER1 |
| 223 | BTBD6 |
| 224 | PSMD13 |
| 225 | DCUN1D3 |
| 226 | COMMD6 |
| 227 | WDR5 |
| 228 | ASB13 |
| 229 | PSMD12 |
| 230 | FBXL22 |
| 231 | COPS8 |
| 232 | KLHL9 |
| 233 | ASB12 |
| 234 | PSMB8 |
| 235 | PSMB10 |
| 236 | UBD |
| 237 | UBA52 |
| 238 | PSMB11 |
| 239 | KBTBD13 |
| 240 | ASB14 |
| 241 | KLHL41 |
| 242 | PSMB9 |
| 243 | KCTD7 |
| 244 | DPP3 |
| 245 | GAN |
| 246 | FBXO17 |
| 247 | PSMB3 |

***Table S2.*** *PH test for 32 candidate genes*

| **candidate genes** | **chisq** | **df** | **p** |
| --- | --- | --- | --- |
| ASB9 | 1.805038 | 1 | 0.1791046 |
| PSME4 | 0.012222 | 1 | 0.9119707 |
| DCUN1D5 | 0.4654297 | 1 | 0.4950973 |
| CUL4A | 0.1705783 | 1 | 0.6795984 |
| PSMB9 | 0.7614023 | 1 | 0.38289 |
| TULP4 | 2.433785 | 1 | 0.1187459 |
| PSMD14 | 0.9217297 | 1 | 0.3370212 |
| UCHL3 | 0.3112657 | 1 | 0.5769046 |
| WDR5 | 0.7951064 | 1 | 0.3725605 |
| PSMA7 | 0.0719308 | 1 | 0.788546 |
| COMMD7 | 1.4191496 | 1 | 0.2335434 |
| PSMC2 | 0.2483069 | 1 | 0.6182697 |
| SOCS2 | 5.4053672 | 1 | 0.0200749 |
| COPS7B | 0.1701165 | 1 | 0.6800083 |
| PSMD12 | 0.0477292 | 1 | 0.8270628 |
| PSMB2 | 0.0764389 | 1 | 0.7821827 |
| UBA52 | 1.5783451 | 1 | 0.2089992 |
| FBXL3 | 1.8147158 | 1 | 0.1779436 |
| DCAF13 | 0.0985388 | 1 | 0.7535902 |
| RBBP7 | 1.3768115 | 1 | 0.240645 |
| PSMB5 | 3.8942495 | 1 | 0.0484517 |
| PSMA1 | 1.8373443 | 1 | 0.1752625 |
| COPS6 | 1.0934719 | 1 | 0.2957032 |
| DCAF5 | 0.4702297 | 1 | 0.4928815 |
| FBXL5 | 1.1244149 | 1 | 0.2889698 |
| CDKN1A | 0.8188188 | 1 | 0.3655257 |
| FBXO22 | 6.635568 | 1 | 0.0099962 |
| UBB | 0.0756209 | 1 | 0.783322 |
| DCAF11 | 0.3945579 | 1 | 0.5299133 |
| WSB2 | 1.5628609 | 1 | 0.2112468 |
| FBXO32 | 0.4257005 | 1 | 0.5141062 |
| EPAS1 | 0.0811671 | 1 | 0.775722 |

***Table S3.*** *PH test of risk score and clinical factors*

| **variate** | **chisq** | **df** | **p** |
| --- | --- | --- | --- |
| risk score | 1.114353592 | 1 | 0.291137528 |
| age | 2.178219 | 1 | 0.139976362 |
| T stage | 0.89147913 | 2 | 0.64035052 |
| N stage | 3.810986833 | 2 | 0.148749229 |
